# Potential of Carbon-Based Nanocomposites for Dental Tissue Engineering and Regeneration

**DOI:** 10.3390/ma14175104

**Published:** 2021-09-06

**Authors:** Moon Sung Kang, Hee Jeong Jang, Seok Hyun Lee, Ji Eun Lee, Hyo Jung Jo, Seung Jo Jeong, Bongju Kim, Dong-Wook Han

**Affiliations:** 1 Department of Cogno-Mechatronics Engineering, College of Nanoscience and Nanotechnology, Pusan National University, Busan 46241, Korea; mskang7909@gmail.com (M.S.K.); h78crom@naver.com (H.J.J.); joe1026@naver.com (S.H.L.); 2 Department of Optics and Mechatronics Engineering, College of Nanoscience and Nanotechnology, Pusan National University, Busan 46241, Korea; jelee7339@gmail.com (J.E.L.); lisa0245@naver.com (H.J.J.); 3 GS Medical Co., Ltd., Cheongju-si 28161, Korea; eric.jeong@gsmedi.com; 4 Dental Life Science Research Institute/Innovation Research & Support Center for Dental Science, Seoul National University Dental Hospital, Seoul 03080, Korea

**Keywords:** carbon nanomaterial, osseointegration, osteogenesis, surface functionalization, antimicrobial activity

## Abstract

While conventional dental implants focus on mechanical properties, recent advances in functional carbon nanomaterials (CNMs) accelerated the facilitation of functionalities including osteoinduction, osteoconduction, and osseointegration. The surface functionalization with CNMs in dental implants has emerged as a novel strategy for reinforcement and as a bioactive cue due to their potential for mechanical reinforcing, osseointegration, and antimicrobial properties. Numerous developments in the fabrication and biological studies of CNMs have provided various opportunities to expand their application to dental regeneration and restoration. In this review, we discuss the advances in novel dental implants with CNMs in terms of tissue engineering, including material combination, coating strategies, and biofunctionalities. We present a brief overview of recent findings and progression in the research to show the promising aspect of CNMs for dental implant application. In conclusion, it is shown that further development of surface functionalization with CNMs may provide innovative results with clinical potential for improved osseointegration after implantation.

## 1. Introduction

Up to now, metal and metal alloy composites, including titanium, gold, stainless steel, and cobalt-chromium, have been utilized for dental implants due to their toughness, shear/fracture-resistance, and noncorrosive property [1,2,3,4,5]. Despite their superior mechanical characteristics, low biocompatibility has become a major concern. Toxic effects caused by ions released from metallic implants induce adverse tissue reactions that lead to a low success rate in long-term clinical applications [6]. Furthermore, metal-based dental implants need a long time to be integrated with natural bone (three to six months) owing to their non-bioactive nature that leads to low cytocompatibility and osseointegration debasement [7,8]. Furthermore, with recent advances in personalized and biofunctional dental implants, the conventional metal-based materials hold the faintest hope for three-dimensional (3D) printability, antibacterial properties, and drug delivery capacity [9,10].

While conventional dental implants focus on mechanical properties, recent advances in functional materials accelerated the facilitation of functionalities including osteoinduction, osteoconduction, and osseointegration. Osteoinduction is the process that stimulates immature cells toward preosteoblasts to start the bone healing process. Osteoconduction means that new bone grows on a material surface. Osseointegration means the facilitation of stable anchorage by bone-to-implant contact which is achieved by high osteoinduction and osteoconduction properties [11]. Novel composite materials have been employed as a powerful tool for the alteration of physicochemical and biological properties of dental implants that allows preferred bioactivity and reducing side effects. Especially nanomaterial-based surface functionalization offers several advantages, including (i) controllable micron/nanometer-sized topography, (ii) exceptional reactivity by high surface–volume ratio, (iii) unique cell-matrix interaction, and (iv) mechanical reinforcement, which regulate bone cell behaviors and improve mechanical properties of the dental implant [12]. Nanomaterial-functionalized surfaces highly affect cell-matrix interaction, endowing cells facilitation including survival, differentiation capability, and activity of cells. Placement of dental implants on bone tissue activates the cellular events that lead to the formation of new bone directly on the implant surface [13]. From a clinical perspective, facilitation of bone gain, which is promoted by biochemical activities of nanomaterials, is recently highlighted for successful surgery and implant rehabilitation [14,15,16]. Furthermore, tailored control of cellular behaviors offers the possibility on orthodontic treatment such as unilateral condylar hyperplasia [17]. Therefore, nanomaterial-modified surface chemistry and topography are known to activate direct cell-matrix contact to stem cells and precursor cells, leading to higher proliferation and differentiation rate into osteogenic lineages by upregulation of osteogenic genes [18,19,20].

**Figure 1:**
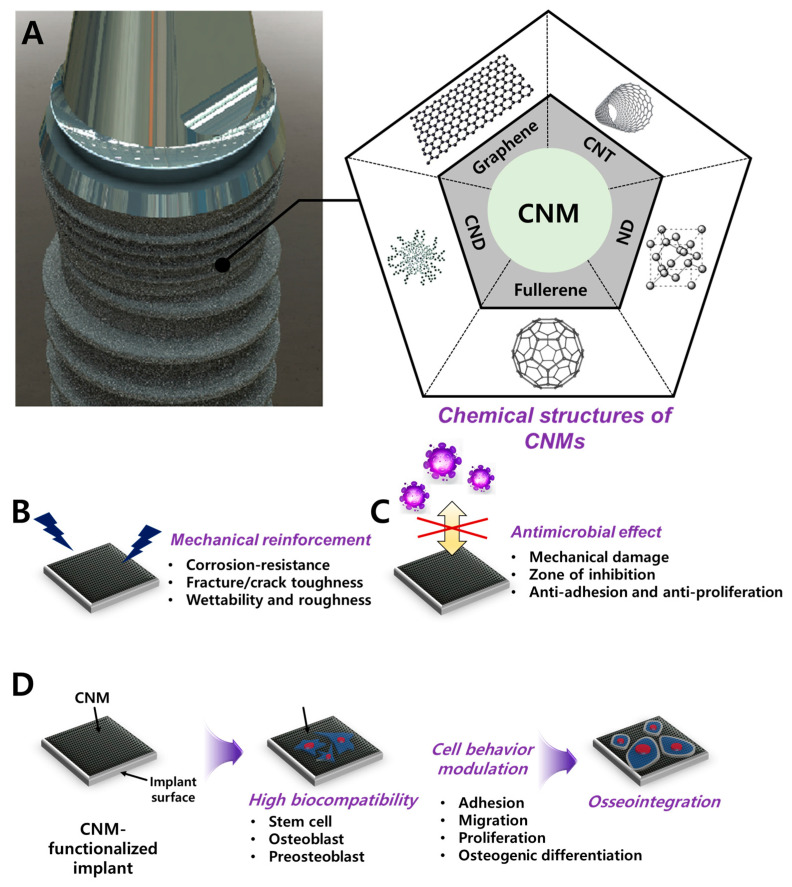
Schematic diagram of CNM functionalization on dental implants for dental tissue engineering and regeneration. (**A**) Graphic of CNM-functionalized implants and chemical composition of the CNM family, including graphene, CNT, CND, fullerene, and ND. Enhanced properties were demonstrated, such as (**B**) mechanical reinforcement, (**C**) an antimicrobial effect, and (**D**) osseointegration.

Carbon nanomaterials (CNMs) can be divided into carbon nanodot (CND), graphene (G) and its derivatives (graphene oxide; GO, reduced graphene oxide, rGO), fullerene, carbon nanotube (CNT), and nanodiamond (ND) (Figure 1). Over the past decade, CNMs are the most highlighted nanomaterials (NMs) in various fields such as aerospace, space, electricity, electronics, and optics. CNMs have revolutionized the biomedical field with antibacterial paper [21,22], targeted drug delivery [23,24,25], in vitro/in vivo bioimaging [26,27,28], tissue engineering scaffolds [29,30], and dental/orthopedic implants [31,32,33], with their extraordinary inherent properties.

**Figure 2:**
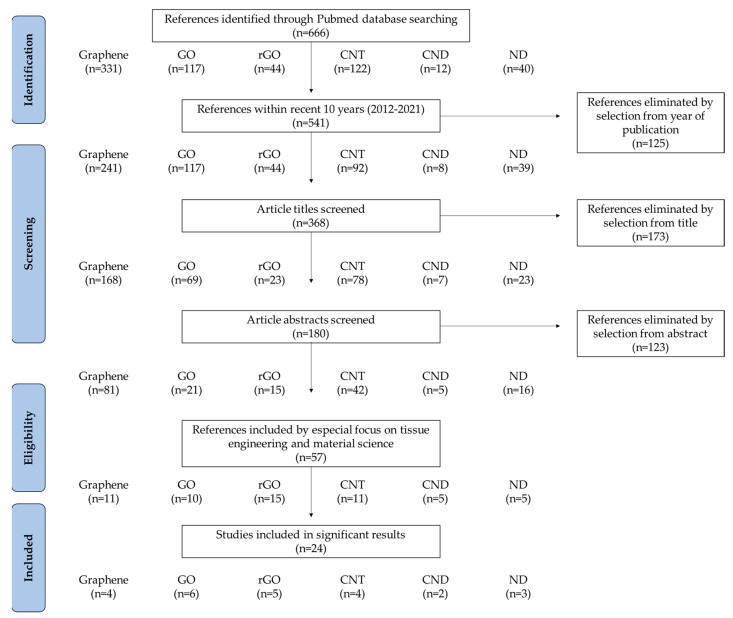
PRISMA flow diagram denoting literature search criteria.

In this review, we discuss the advances in novel dental implants with CNMs in terms of tissue engineering, including material combination, coating strategies, and functionalities (Table 1). Recent studies on CNM functionalization for dental application were sorted by a PRISMA flow diagram (Figure 2). We present a brief overview of recent findings and progression in the research to show the promising aspect of CNMs for dental implant application.

## 2. Physicochemical Properties and Bioapplications of CNMs

CNMs have been highlighted for biomedical applications owing to their extraordinary optical properties [34], the reactive oxygen species (ROS) quenching property, relatively low toxicity [35], ease of chemical modification [36], and chemical stability [37]. The detailed characteristics are altered by various chemical structures of CNMs.

G is composed of sp^2^ hybridized carbon atoms, and their electrons participate in aromatic conjugated domains [38]. GO is a highly oxidative form of G which is generally obtained by oxidation of graphite in a mixture of strong acid and oxidizing agent and is easily water-dispersible. GO features an amphiphilic structure comprising both hydrophobic parts from pristine graphite and a hydrophilic part with oxygen-containing functional groups such as hydroxyl, epoxy, carbonyl, and carboxyl groups on the basal plane and at the edge [39]. The specific chemical structure of GO endows unique properties such as affinity for aromatic rings, water dispersibility, and biocompatibility [39]. Meanwhile, rGO maintains graphene domains with structural defects while remaining residual oxygen-containing groups on the surface of the sheets promote protein adsorption and cell adhesion [40,41,42]. CND is a zero-dimensional carbon-based tens of nanometer-sized material, which is isolated from a single-walled carbon nanotube (SWCNT) [43]. CNTs have long and hollow tube structures made of one or multiple layers of graphene, which are called SWCNT and multi-walled CNT (MWCNT), respectively. Except for the large surface area and great mechanical strength, high electroconductivity enables a bioelectric system immobilizing DNA and proteins on the surface of CNTs [44]. The antioxidant activity against thermal or photodegradation of polymers and the UV stabilization activity, which is beneficial for exposure to UV and oxygen, are other benefits of CNTs [45,46].

Fullerene contains 60 carbon atoms with C_5_-C_5_ single bonds forming pentagons and C_5_-C_6_ double bonds forming hexagons [47]. The fullerenes can act as a free radical scavenger with a delocalized π double bond system allowing quenching of various free radicals more efficiently than conventional antioxidants [48]. Fullerenes have been utilized for photodynamic therapy, neuroprotection, apoptosis, drug and gene delivery, and diagnostic purposes [48]. ND is composed of an sp^3^ hybridized carbon core and surface functionalized with fewer but various moieties including phenols, pyrones, and sulfonic acid, as well as carboxylic acid groups, hydroxyl groups, and epoxide groups [49]. The chemical inertness of core and kinds of functional moieties enables noncovalent or covalent attachment of drugs or biomolecules, material composition, or hybridization for biomedical applications, especially for in vivo and in vitro bioimaging [50].

## 3. Biocompatibility of CNMs

The extensive potentials of CNM for biomedical application have been highlighted, including antibacterial [51,52], cell adhesion and proliferation [53,54], inducing osteogenic [55,56], osteoconduction [57,58], and osseointegration effects [59]. However, biocompatibility, which often shows contradictory or inconclusive results, has issues that should be elucidated. The biocompatibility of CNMs often time-, size-, and dose-dependently works, however, it varies by raw materials, fabrication methods, and physicochemical functionalization [60,61,62]. Since it is difficult to draw accurate conclusions, we intend to provide guidelines for later studies by comparing relevant studies.

GO’s dose-dependent cytotoxicity on bone marrow-derived stem cells (BMSCs) and assessed toxicity mechanisms were investigated [63]. GO significantly inhibited cell viability at ≥2.5 µg/mL by interrupting membrane integrity. At the same concentration, cell apoptosis was one-and-a-half-fold increased but did not hinder the cell proliferation cycle significantly. Furthermore, ≥2.5 µg/mL of GO induced intracellular ROS generation, inducing ROS-associated damage, and caused cell dysfunction which was assessed by mitochondria membrane potential (MMP) loss. Western blotting showed upregulation of Cleaved Caspase-3, LC3-II/I, and Beclin-1 and downregulation of Bcl-2 and Caspase-3, indicating that GO-mediated cytotoxicity is related to mitochondrial autophagy and triggering cellular apoptosis. The hemolytic and cytotoxic effects of GO, which are synthesized in various methods, showed varying results according to their sizes, particulate states, surface charges, and oxygen contents [64]. Hemolysis and morphologies of red blood cells (RBCs), intracellular ROS generation, and fibroblast viability were significantly different according to the fabrication methods, suggesting that the particulate state of G materials has a profound impact on cytotoxicity. The cytotoxicity and genotoxicity of different CNMs are proven to be material-specific and cell-specific with a general trend for biocompatibility (ND > carbon powder > MWCNT > SWCNT > fullerene) [50]. For example, macrophages are more cytotoxic than neuroblastoma cells, and CNT and MWCNT tend to cause DNA damage in mouse embryonic stem cells by ROS generation [50]. NDs possess minimal cytotoxicity because their chemical inertness does not release any toxic chemicals and round morphologies [65]. Carboxylated NDs were shown to not exhibit cytotoxicity or genotoxicity on human cell lines including liver, kidney, intestine, and lung cell lines, which are major accumulation organs after the nanoparticles are injected [66]. On the other hand, fullerene shows significant cytotoxicity mainly contributing to ROS generation. Fullerenes under ambient water conditions can generate superoxide anions that are responsible for membrane damage and subsequent cell death [67].

For clinical usages including drug delivery, bioimaging, biosensing, and other theragnostic applications, in vivo toxicity of CNMs has been intensively studied. To understand the potential threat of CNMs in the body, biodistribution and accumulation mechanisms should be elucidated. The accumulation of GO in mouse lung induced oxidative stress by an increase of mitochondrial respiration and activated inflammatory and apoptotic pathways [68]. On the other hand, surface functionalization and chemical modification have been introduced to enhance the biocompatibility and biofunctionality of G materials [69,70,71]. The PEGlyated GO and rGO were developed for oral and intraperitoneal (i.p.) injection, and the biodistribution was investigated [72]. After seven days, oral administration could not be adsorbed by organs and rapidly excreted, however, i.p.-administered PEGlyated GO and rGO were accumulated most highly in the liver and spleen but were finally engulfed by phagocytes in size- and surface coating-related manner. The results indicated that no significant toxicity was found in serum biochemistry, complete blood panel test, and histological analysis, indicating that PEGylation can facilitate biocompatibility of G materials. In a similar study, intravenous (i.v.) injected G quantum dots (GQDs) did not exhibit to vital organs of rats, although slight reduction of platelets and monocyte and eosinophil fractions occurred, which were soon normalized [73]. After respiratory administration, CNT remains in the lungs for months or even years and is eliminated through the gastrointestinal (GI) tract. It does not cross the pulmonary barrier or get absorbed in the GI tract [74,75]. A single intratracheal instillation of SWCNT triggered epithelial granulomas and interstitial inflammation, developing peribronchial inflammation and necrosis [76].

**Table 1:** Recent studies on CNMs for dental implant application.

Classification of CNM	Conjugation/Combination/Modification Material	Physicochemical Advances	Osteogenic/Antimicrobial Activities	Biological Evaluation (Species)	Reference
Graphene	Zinc oxide nanocomposite coating on the acrylic tooth	-	Antimicrobial and nontoxicity on human cell	In vitro (*S. mutans*, HEK-293 cell)	[32]
	G nanoplatelet coating	-	Antimicrobial effect	In vitro (*S. aureus*)	[77]
	G-doped PMMA	-	Increased bone formation indexes (NBF, BMI, LBD, BIC, BAIT, and BAOT)	In vivo (rabbit)	[78]
	Composite with Y-Zr ceramics	Increased density, Vickers hardness, bending strength, fracture toughness, and wettability	-	-	[79]
Graphene oxide	GO/3Y–ZrO_2_ composite	Reduced friction coefficient, wear rate, surface roughness. Increased wetting property.	Increased cell adhesion, proliferation, and ALP activity.	In vitro (MC3T3-E1 cell)	[80]
	NT/GO-PEG-PEI/siRNA	-	Enhanced cell adhesion, proliferation, uptake/knockdown efficiency, osteogenic gene expression, ALP activity, collagen secretion, ECM mineralization, and in vivo osseointegration	In vitro (MC3T3-E1 cell) and in vivo (mouse)	[81]
	MH-loaded GO film on Ti	-	Prevention and therapeutic effect on peri-implantitis	In vivo (Beagle dog)	[82]
	Nano GO-coated Ti/SLA surface	Rough and irregular surface, wettability, protein adsorption	Enhanced cell proliferation, cell area, focal adhesion formation, mineralization, and osteogenic gene expression via the FAK/MAPK signaling pathway	In vitro (rBMSC) and in vivo (SD rat)	[83]
	MMP-2/SP-loaded GO/Ti	Enhanced roughness and wettability	MMP-2/SP delivery facilitated new bone formation	In vivo (mouse)	[84]
	GO/PEEK	Surface roughness and wettability	Antibacterial ability, enhanced cell viability, proliferation, ALP activity, mineralization nodule formation, osteogenic gene expression	In vitro (MG-63 cell, *E. coli* and *S. aureus*)	[85]
Reduced graphene oxide	DCP-rGO composites	Controllable hybridization ratio	Cell proliferation, ALP activity, and mineralization	In vitro (MC3T3-E1 cell)	[86]
	Dex/GO-Ti and Dex/rGO-Ti	Dex-loading capacity	Cell proliferation, osteogenic gene expression, and mineralization	In vitro (rBMSC)	[87]
	Dex/rGO-coated Ti_13_Nb_13_Zr	Enhanced wettability and fatigue property	Enhanced cell viability, mineralization, and osteogenic gene upregulation	In vitro (MC3T3-E1 cell)	[88]
	rGO/FHAp composites	Enhanced mechanical strength (GPa, MPa), ion dissolution time	Enhanced cell proliferation, ALP activity, and anti-adhesion/proliferation on bacteria	In vitro (MC3T3-E1 cell and *S. mutans*)	[89]
	rGO-coated Ti6Al4V alloy	-	Enhanced cell viability, adhesion, proliferation, mineralization nodule formation, ALP activity, and osteogenic gene expression	In vitro (MC3T3-E1 cell)	[90]
Carbon nanodot	Nitrogen-doped CND/HA composite		Enhanced cell proliferation, ALP activity, mineralization nodule formation, and osteogenic gene expression. Bone regeneration in zebrafish jawbone model	In vitro (MC3T3-E1 cell) and in vivo (zebreafish)	[91]
	CND/chitosan/HAp composite	Photothermal effect	Cell adhesion and osteogenesis, no lobulated neutrophils, osteocyte proliferation, tumor cell killing effects, and antibacterial effects	In vitro (rat BMSC, *S. aureus* and *E. coli*) and in vivo (mouse)	[92]
Carbon nanotube	MWCNT-reinforced HAp coated Ti6Al4V implant	Cost-effective and rapid coating via electrophoresis. No microcracking, increased bond strength, and peeling resistance.			[93]
	MWCNT-reinforced HAp/316L SS implant	High corrosion protection and corrosion current density	Antibacterial effects and nanoflake morphology for enhancing bioactive potential	In vitro (*B. subtilis*, *S. aureus*, *S. flexneri* and *E. coli*)	[94]
	Cu-HAp/MWCNT composite coating on 316L SS implant	High corrosion resistance	Antibacterial effect, maintained cell viability, hemolytic activity	In vitro (human osteoblast, human RBC, *B. subtilis*, *E. coli*, *S. aureus*, and *S.mutans*)	[95]
	Nano HAp/MWCNT coated stainless steel	Increased surface roughness	No damage on the cellular membrane and enhanced expression of osteogenic markers.	In vitro (MG-63 cell)	[96]
Nanodiamond	ND/amorphous carbon composite	-	Enhanced fibronectin expression, attachment, proliferation, differentiation, calcium deposition, and ALP activity.	In vitro (EPC)	[97]
	Icariin-functionalized ND composite	-	Icariin delivery, enhanced cell viability, particle uptake, ALP activity, calcium deposition, and osteogenic marker upregulation.	In vitro (MC3T3-E1 cell)	[98]
	Mg-nanodiamond composite	pH buffering, corrosion resistance, chemical passivation	Moderate cell viability	In vitro (L-929 cell)	[99]

**Abbreviations:** alkaline phosphatase (ALP), *Bacillus subtilis* (*B. subtilis*), bone area inner threads (BAIT), bone area outer threads (BAOT), bone marrow mesenchymal stem cell (BMSC), bone mature index (BMI), bone-to-implant contact (BIC), dexamethasone (Dex), dicalcium phosphate (DCP), *Escherichia coli* (*E. coli*), extracellular matrix (ECM), endothelial progenitor cells (EPC), fluorhydroxyapatite (FHAp), hydroxyapatite (HAp), lamellar bone direct contact (LBD), minocycline hydrochloride (MH), morphogenetic protein-2 (MMP-2), new bone formation(NBF), Polyetheretherketone (PEEK), polyethylene glycol (PEG), polyethyleneimine (PEI), poly(methyl-methacrylate) (PMMA), red blood cell (RBC), sandblasting and acid etching (SLA), *Shigella flexneri* (*S. flexneri*), siRNA (small interfering), *Staphylococcus Aureus* (*S. aureus*), *Streptococcus mutans* (*S. mutans*), substance P (SP), rat bone marrow mesenchymal stem cell (rBMSC), titania nanotube (NT), titanium (Ti), yttria-zirconia (Y-Zr), 3Y (three-mol yttria-stabilized).

## 4. CNMs for Dental Application

**Figure 3:**
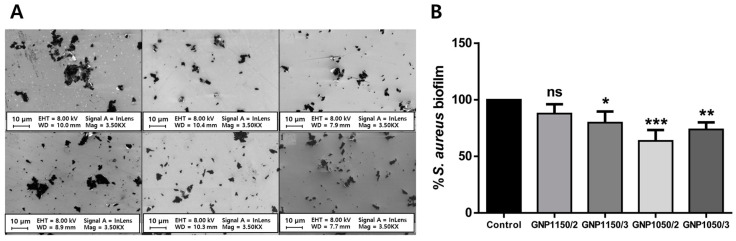
(**A**) Field emission scanning electron microscopy (FE-SEM) on G nanoplatelets fabricated with different conditions. (**B**) Biofilm inhibition for *S. aureus*. The asterisks denote significant difference compared to control (*: *p* < 0.05, **: *p* < 0.01, and ***: *p* < 0.001). ‘ns’ refers not significant. Data reproduced from Ref. [77]. Copyrights MDPI 2020.

Antibacterial materials are widely used in dentistry and effectively alleviate the risk of inflammation of implantation. A wide range of antibacterial materials including antibiotics [100,101], metal ions [102,103], and quaternary ammonium compounds [104,105] have been introduced for the prevention of attachment and proliferation of microbes on implant surfaces. However, these materials are hurdled due to high cost, complex processing, low biocompatibility, and environmental problems. G materials chemically and physically interact with bacterial membrane and morphological alteration, membrane integrity destruction, inducing RNA and intracellular materials leakage [51]. These phenomena can be explained by physical damage of sharp edges of G materials and lipid peroxidation initiated by the oxidative ability of G materials [106]. Zinc oxide graphene nanocomposites (GZNC) were coated on acrylic tooth implants. Oral biofilms that accumulate on implant surfaces are the most common cause of dental implant failure. *S. mutans* is considered one of the main pathogens that induce the development of secondary dental caries [107]. A recent study reported that surface topography and chemical composition reinforced by nanomaterial incorporation could play an important role in inhibiting bacterial adhesion and biofilm formation [107]. The GZNC coating layer showed a significant reduction in the biofilm of *S. mutans*, which is one of the primary etiological agents in dental caries. The results indicated that GZNC is nontoxic to human embryonic cell line HEK-293 cells, suggesting the biocompatibility and effectivity of GZNC coating for dental implant application [32]. G nanoplatelets were coated on a Ti implant surface to prevent peri-implant disease (Figure 3A) [77]. The direct contact of the implant surface and surrounding tissues often induces microbial infection, leading to peri-implant disease such as mucositis or peri-implantitis [108,109]. G nanoplatelets have no oxygen-containing functional groups on the basal plane, therefore, they do not generate ROS while inhibiting microbial adhesion on surfaces [110]. The crystal violet binding assay showed that G nanoplatelet-coated surfaces significantly decrease the formation of an *S. aureus* biofilm, and the degree of inhibition was different according to G nanoplatelet preparation methods (Figure 3B) [77]. On the other hand, in vivo osseointegration of G-coated implants has been elucidated. G-doped PMMA were coated onto the implant, and implantation efficacy was tested by micro-CT and histomorphological assays in rabbits [78]. Bone formation indexes, including NBF, BMI, LBD, BIC, BAIT, and BAOT, were compared between pristine PMMA and G-doped PMMA implants. Acid fuchsin and toluidine blue staining of bone samples showed newly formed bone in implant–bone contact, and mature bone was observed after 30 days. BAIT, BOAT, and VOI were superior in the G-doped PMMA implant compared to pristine PMMA, meaning better osseointegration. These results suggest that a G-doped implant induces no adverse tissue reaction and effectively supports selective bone regeneration in vivo.

Focusing on osseointegration and mechanical reinforcement, GO is more frequently incorporated as an implant coating material. GO has a large number of functional groups, including epoxy, carbonyl, carboxyl, and hydroxyl groups, bound on the basal planes and edges. This makes GO hydrophilic, readily dispersible in water and solvents, and easily modified to make composite materials [111]. Moreover, the functional moieties facilitate cell-matrix interaction between the implant surface and surrounding cells, enhancing cellular behaviors including adhesion, proliferation, migration, and differentiation into specific lineages [29,111,112,113]. GO was incorporated on Y-Zr ceramics for dental implants application [79]. Y-Zr is a ceramic which is a tetragonal crystal from zirconium dioxide made stable at room temperature and added yttrium oxide. The surface functionalization of GO mechanically reinforced the Y-Zr implant, giving increased relative density, Vickers hardness, bending strength, fracture toughness, and wettability. On the other hand, GO coating on Y-Zr has proven to give biological properties and osteogenesis-inducing capability. The effects of GO on mechanical and biological properties of 3Y-ZrO_2_/GO composite dental implants were investigated [80]. The addition of GO reduced the friction coefficient, wear rate, and surface roughness due to self-lubricating properties. Furthermore, the hydroxyl groups of GO gave an increment of wetting property. In an in vitro assay with MC3T3-E1 preosteoblasts, GO increased adhesion, proliferation, and ALP activity (i.e., one of the early osteogenic markers).

**Figure 4:**
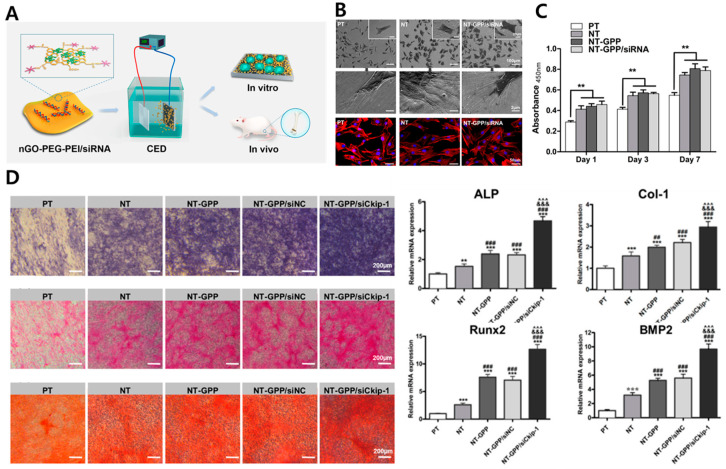
GO-PEG-PEI/siRNA composite for facilitation of osteogenesis (**A**) schematic diagram of GO-PEG-PEI/siRNA composite. (**B**) Optical and fluorescence microscopy of MC3T3-E1 cells cultured on Wet polished Ti (PT), NT, and NT-GPP/siRNA substrate. (**C**) Proliferation assay (**D**) ALP activity (purple), collagen secretion (pink), and ECM mineralization (orange), and osteogenic gene expression of cells cultured on PT, NT, and NT-GPP/siRNA substrate. Each symbol represents significant difference between groups (** and *** *p* < 0.01 and 0.001 vs. PT. ## and ### *p* < 0.01 and 0.001 vs. NT. &&& *p* < 0.001 vs. NT–GPP. ^^^ *p* < 0.001 vs. NT–GPP/siNC). Data reproduced from Ref. [81]. Copyrights ACS 2017.

Meanwhile, GO can be used as a drug delivery system owing to its wide surface area, chemical and mechanical constancy, sublime conductivity, and biocompatibility [114]. The dual usage of GO was investigated as a siRNA delivery system and osteogenesis-inducing component for a Ti implant (Figure 4A) [81]. The siRNA showed great potential for bone regeneration of the implant with its tissue targetability and high specificity [115,116]. Osteogenic efficacy of the NT/GO-PEG-PEI/siRNA composite was evaluated both in vitro (MC3T3-E1 cell) and in vivo (mouse). The GO-PEG-PEI/siRNA composite enhanced cell adhesion, proliferation, uptake/knockdown efficiency of siRNA, an increase of ALP activity, collagen secretion, and ECM mineralization (Figure 4B–D). Van Gieson staining and EDX scanning of mouse bone cylindrical implants after one month of implantation revealed enhanced osseointegration and new bone formation. The MH-loaded GO films on implant abutment were investigated for peri-implantitis treatment [82]. In this study, GO film was also dual-used for osseointegration and MH-delivery system. MH is a tetracycline antibiotic that exhibits antibacterial properties against *S. aureus*, *E. coli*, and *S. mutans* [117,118]. The MH-loaded GO implants were fabricated and implanted in a peri-implantitis model in beagle dogs. The micro-CT tomography and histological evaluation demonstrated not only the osseointegration effect of MH-loaded GO implants but also the prevention and therapeutic effect for peri-implantitis mainly due to their excellent antibacterial activity.

Nano GO-coated Ti implants can mediate osteogenesis by involving FAK/P38 signaling pathways [83]. Due to its nano-topographical characteristics with GO, the surface got rough and irregular, increasing protein adsorption and wettability. An in vitro assay revealed that nano GO-coating enhanced cell proliferation, cell area, focal adhesion formation, mineralization, and osteogenic gene expression via modulation of FAK and its downstream MAPK/P38 signaling pathway, which suggests that nano GO could be an excellent material for implant surface functionalization. BMP-2 is a well-known growth factor for inducing osteogenesis of stem cells, and SP is reported to be involved in the regulation of inflammation, wound healing, and angiogenesis [119,120,121,122]. The BMP-2 and SP delivery strategy with a GO coating on a Ti implant were introduced for better osteoconduction and osseointegration [84]. BMP-2/SP-loaded GO-coated Ti ring orthopedic implants were implanted in mice, and histomorphometry was assessed after eight weeks, indicating that 4 mm^2^ of new bone formed compared to bare Ti implants. Meanwhile, GO-PEEK orthopedic implants featuring antibacterial and osteogenic capabilities were investigated [85]. GO-PEEK composites inhibited the growth of *E. coli* but did not show inhibition effects on *S. aureus*. However, MG-63 cells showed enhanced cell viability, proliferation, ALP activity, mineralization nodule formation, and osteogenic gene expression including osteocalcin (OCN), RUNX2, and collagen type I, suggesting osteogenesis facilitation of GO.

**Figure 5:**
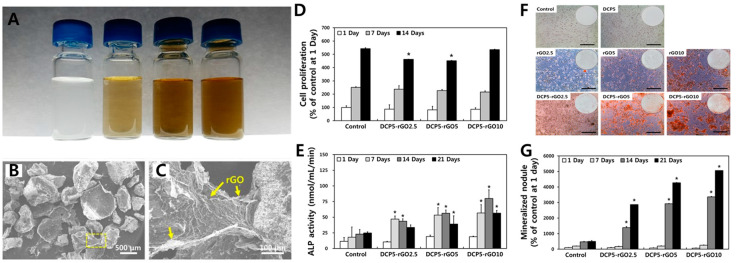
DCP-rGO hybrid composites for osteogenesis of MC3T3-E1 preosteoblasts. DCP and rGO at concentration ratios of 5:2.5, 5:5, and 5:10 μg/mL were designated as DCP5-rGO2.5, DCP5-rGO5, and DCP5-rGO10, respectively. (**A**) (From the left) Digital images of phosphate-buffered saline (PBS), rGO2.5, DCP5-rGO5, and DCP5-rGO10. (**B**) Low and (**C**) high magnification SEM of DCP-rGO hybrid composites. (**D**) Proliferation, (**E**) ALP activity, (**F**) macroscopic images of ARS staining, and (**G**) mineralization nodules of MC3T3-E1 cells cultured with DCP-rGO composites. An asterisk (*) denotes significant difference compared to the control of the same day. Data reproduced from Ref. [86]. Copyrights MDPI 2017.

**Figure 6:**
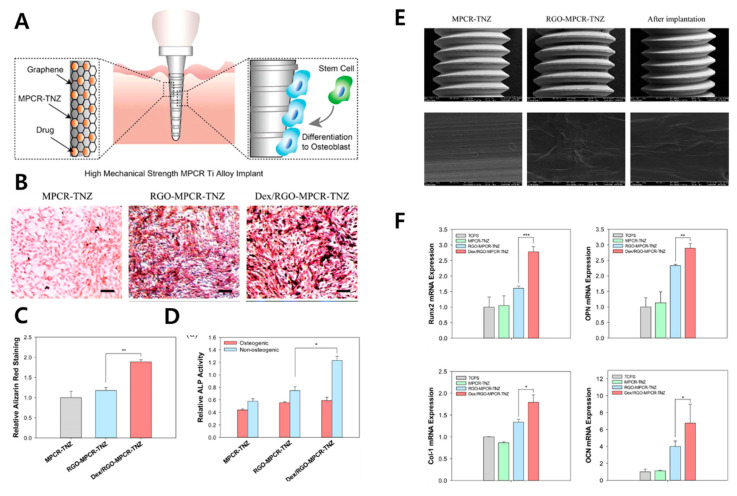
Dex/rGO-MPCR-TNZ composite. (**A**) Schematic diagram of Dex/rGO-MPCR-TNZ implant. (**B**) ARS staining microscopy and (**C**) quantification. (**D**) ALP activity. (**E**) SEM of each implant sample before and after implantation. (**F**) Relative osteogenic gene expression. The asterisks denote significant difference compared to control (*: *p* < 0.05, **: *p* < 0.01, and ***: *p* < 0.001). Data reproduced from Ref. [88]. Copyrights ACS 2015.

rGO has chemical and physical nature different from those of G and GO. The structural deficiency with residual functional moieties can be sharply tailored. For example, surface oxygen content and degree of reduction have significant influence on protein adsorption and effects on cellular behaviors [123]. rGO is often hybridized with bioceramics which are believed to be a promising candidate for bone substitutes due to their biocompatibility and strength. The DCP-rGO hybrid composites for osteogenic effects on MC3T3-E1 preosteoblasts were evaluated (Figure 5) [86]. DCP-rGO composites showed irregular granule-like micron-sized structure. Enhanced ALP activity and calcium nodule deposition of MC3T3-E1 cells with DCP-rGO composites indicated that DCP-rGO composites can accelerate osteogenic differentiation by the synergistic effects of rGO and DCP. The osteogenic effect of Dex (i.e., a glucocorticoid drug promoting osteogenic differentiation of stem cell and progenitor cells [124])-loaded GO or rGO-Ti composites on BMSCs were evaluated [87]. Dex loading amounts and release profile were significantly increased in both GO-Ti and rGO-Ti compared to those of pristine Dex. Therefore, proliferation, ALP activity, mineralization nodule formation, expression of osteopontin (OPN), and OCN were increased, showing the promoting effects of highly delivered Dex on BMSCs. Multipass caliber-rolled (MPCR) Ti_13_Nb_13_Zr dental implants functionalized with Dex-loaded rGO (Dex/rGO-MPCR-TNZ) (Figure 6A) were developed [88]. The MPCR process is used for the introduction of an ultra-fine-grained structure with rGO on a Ti surface that enhances wettability and fatigue property. In vitro assays on MC3T3-E1 cells showed promoting effects on cell viability, mineralization, and osteogenic gene upregulation (RUNX2, OPN, OCN, and collagen type Ι), which could contribute to the osteogenic effect and Dex delivery efficiency of rGO (Figure 6B–F). On the other hand, HAp is a natural bioceramic composing the dental enamel and dentine. HAp has high moisture resistance and ideal hardness similar to that of the natural bone matrix [87]. By hybridizing the osteogenic effects of rGO and HAp, rGO/FHAp composite implants were investigated [89]. rGO/FHAp increased implant hardness by 86% and fracture toughness by 137%. Compared to pure HA, FHAp (fluoride partially substituted in HAp) enhanced the chemical stability of the implant. Moreover, rGO/FHAp enhanced proliferation and ALP activity of MC3T3-E1 cells and inhibited adhesion and proliferation of *S. mutans*, suggesting its exceptional osteogenic and antibacterial properties. Another study showed enhanced growth and osteogenic differentiation of MC3T3-E1 cells on rGO-functionalized Ti6Al4V implants [90]. The incorporation of rGO significantly increased cell viability, adhesion, proliferation, mineralization nodule formation, ALP activity, and osteogenic gene expression including RUNX2, OCN, OPN, and bone sialoprotein (BSP). These osteogenic effects of rGO could be attributed to several mechanisms. An rGO coating could affect the microstructure of the surface, protein adsorption, and electrostatic interaction. Moreover, rGO is known to accumulate high amounts of Ca^2+^ by π–π stacks between aromatic rings, which can provide the cells with a favorable osteogenic environment [125].

Although CNDs are rarely utilized for dental implants, recent studies highlighted their exceptional osteogenic properties. CNDs are known to promote osteogenic differentiation in rat BMSCs by upregulating the expression of osteogenic markers, including runt-related transcription factor 2 (RUNX2), ALP, OCN, and bone sialoprotein [126]. The osteogenic potential of nitrogen-doped CNDs conjugated with HAp (NCND/HAp) NPs was assessed and showed significant facilitation of osteoblast behaviors and the zebrafish jawbone regeneration model [91]. Owing to the photoluminescence property of CND, the conjugate exhibited high fluorescence intensity by UV radiation. The NCND/HAp NPs promoted cell proliferation, ALP activity, mineralization nodule formation, and osteogenic gene expression of MC3T3-E1 cells, indicating the osteogenic effects of conjugates. The NPs were endocytosed by cells and made the morphology of the cells clearly visualized by green fluorescence emission. Moreover, in vivo zebrafish jawbone regeneration had a three-fold increase compared to that in the control group, suggesting that CNDs improved bone metabolism effectively by enhancing bone volume and bone mineral density. On the other hand, CND-doped chitosan/HAp (CND/CS/HAp) scaffolds were proven to have the potential to promote bone regeneration and multimodal property with tumor ablation and bacterial eradication by their photothermal activity [92]. The CND/CS/HAp scaffolds were i.p. injected into mice, and 10 min of NIR radiation increased the temperature of CND/CS/HAp scaffolds up to 50 °C at the tumor site. Moreover, H&E staining of the harvested samples showed no lobulated neutrophils, and an increased osteoblast population with more collagen and vessels were formed entirely in the CND/CS/HAp scaffolds.

**Figure 7:**
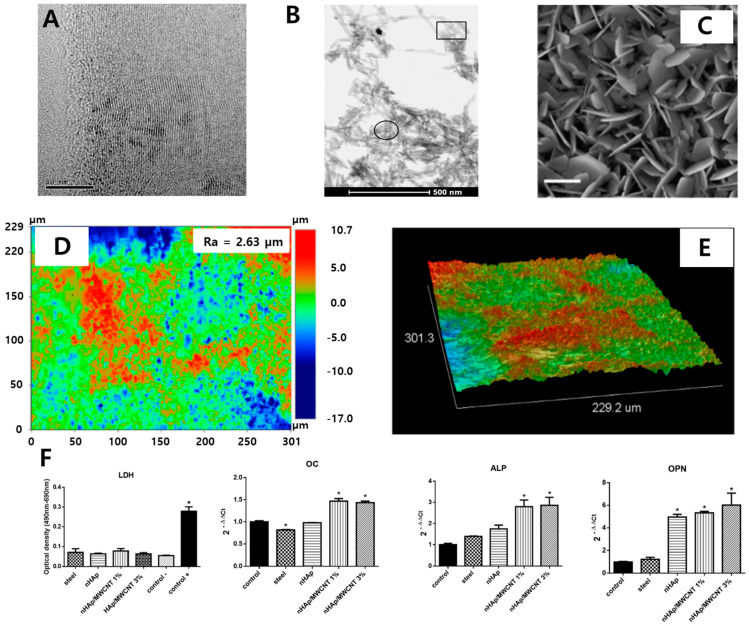
CNT/MNCNT composite-coated 316L stainless steel alloys. (**A**) Transition electron microscopy of MWCNT. (**B**) SEM of nano HAp-deposited MWCNT. The square illustrates the MWCNT covered by nHAp crystals, and the circle illustrates a region containing pure nHAp crystals. (**C**) SEM, (**D**) optical images from profolometry, and (**E**) 3D constructions extracted from profilometry of nanoHAp/MWCNT-deposited 316L stainless steel. (**F**) Intracellular lactate dehydrogenase (LDH) release and expression of osteogenesis-related genes (OC: osteocalcin, ALP, OPN). An asterisk (*) denotes significant difference compared to the control of the same day. Data reproduced from Ref. [96]. Copyrights MDPI 2015.

CNT has been widely utilized for the reinforcement of dental implants due to its outstanding mechanical property, corrosion resistance, osteogenic property, and antibacterial property. MWCNTs are composed of several core-shelled graphene sheets that feature an exceptional Young’s modulus (1000–3000 GPa) and tensile strength (30 GPa) [127,128]. The poor mechanical strength of HA coating layers on the implant can be reinforced by MWCNT. MWCNT-reinforced HAp coated Ti6Al4V implants were developed, and the mechanical characteristics were investigated [93]. The MWCNT/HAp composites were coated on implants by electrophoresis, which features a cost-effective and rapid process. The results indicated no microcracking, increased bond strength between coating layers and implants, and peeling resistance. Furthermore, the corrosion resistance and antibacterial properties of MWCNT/HAp composites were evaluated [94]. HAp/MWCNT-coated 316L SS implants were fabricated by spray pyrolysis and exhibited greater corrosion protection and corrosion current density than pristine 316L SS implants. Moreover, the flower-like nanoflake morphology of the surface induced the necrosis of *B. subtilis*, *S. aureus*, *S. flexneri*, and *E. coli*, suggesting the antibacterial potential of HAp/MWCNT composite coatings. To stimulate the physiological activities of adjacent cells, several essential elements can be substituted into composites. Cu-substituted HAp/MWCNT-coated 316L SS implants were developed, and the biological advantages were assessed. The results suggest that a cytocompatible concentration should be considered to not induce cytotoxicity and hemolysis [95]. The osteogenic potential of HAp/MWCNT was elucidated. Nano HAp/MWCNT coated stainless steel implants were developed, and the in vitro osteogenic properties were evaluated (Figure 7) [96]. The nano HAp/MWCNT coatings significantly increased the expression of osteogenic genes, including OCN, ALP, and OPN of MG-63 cells, indicating that they facilitate both initial and later osteogenic differentiation.

ND coatings and composites have been highlighted for their osteogenic potential and excellent mechanical and tribological performances, as well as enhanced adhesion, growth, and maturation of bone cells [129,130]. The effects of ND/amorphous carbon (ND/a-C) composites containing hydrogel for the facilitation of osteogenesis of EPCs were demonstrated. Fibronectin expression increased on ND/a-C-containing hydrogel, hence attachment and proliferation of EPCs were significantly enhanced. In periods of culture, osteogenic differentiation, including calcium deposition and ALP activity, was promoted, suggesting the osteogenic potential of ND. ND can be utilized as a drug delivery vehicle due to its low toxicity and biocompatibility. To enhance the in vitro osteogenic capacity, icariin, which possesses osteoinductive effects for bone tissue regeneration and is widely used for the treatment of postmenopausal osteoporosis, was incorporated into ND (ICA/ND), and the osteogenic potentials were evaluated [98]. ND efficiently delivered icariin into cells by particle endocytosis. Hence, ALP activity, calcium deposition, collagen secretion, and osteogenic marker regulation were significantly increased in MC3T3-E1 preosteoblasts. On the other hand, Mg is known to be osteoconductive and osteoinductive with physical characteristics similar to those of bone [131]. However, rapid biodegradation and low corrosion resistance have been hurdles for the dental application of Mg. Mg/ND composites were fabricated, and the biodegradation behaviors and cytotoxicity were evaluated [99]. The Mg/ND composites exhibited pH-buffering effects and corrosion resistance by spontaneous formation of a passivation layer, consuming calcium and phosphate ions to form calcium phosphate clusters.

## 5. Conclusions and Future Perspectives

Recent research elucidating the mechanical, antimicrobial, and biological characteristics of CNMs has provided the basis for the potential applications of CNMs as coating agents for bone tissue engineering scaffolds and dental implants. In this review, the biocompatibility improvement, various conjugation/combination/modification materials and methodologies, physicochemical advances, and osteogenic/antimicrobial activities from recent studies have been summarized and discussed. Particularly, it was found that through novel approaches of surface functionalization with CNMs, mechanical stability and biological activity were significantly improved both in vitro and in vivo. Great drug delivery potential of CNMs promises the acceleration of spontaneous healing by higher loading of growth factors, adhesive peptides, and osteogenic signaling molecules into dental implants and scaffolds. Moreover, the unique electrical characteristics of CNMs provide a versatile opportunity to be combined with microelectronics to stimulate, control, and even alter cellular behaviors as well as diagnose and monitor oral and periodontal diseases. The osteogenesis-inducing property of CNMs would play a pivotal role in the full regeneration of damaged dental tissues in the future. Although research in the field of carbon nanoscience is positive, the inherent toxicity and safety of CNMs, large-scale manufacturing, and clinical applications are still challenging. Nevertheless, it is expected that further development of surface functionalization by using CNMs may pave the way for innovative upgrading of dental implants.

## Data Availability

No new data were created or analyzed in this study. Data sharing is not applicable to this article.
